# Antibiotics-induced dysbiosis impacts dendritic morphology of adult mouse cortical interneurons

**DOI:** 10.3389/fnana.2025.1557961

**Published:** 2025-03-07

**Authors:** Mohammed M. Nakhal, Ayishal B. Mydeen, Lydia K. Yassin, Reem Almazrouei, Rasha Alkamali, Mahra Alsulaimi, Rawan I. Elsaleh, Shamsa BaniYas, Shaikha Al Houqani, Farah Al-Marzooq, Maya Hassane, Roman Voitetskii, Yauhen Statsenko, Mushal Allam, Amal Akour, Mohammad I. K. Hamad

**Affiliations:** ^1^Department of Anatomy, College of Medicine and Health Sciences, United Arab Emirates University, Al Ain, United Arab Emirates; ^2^Department of Medical Microbiology and Immunology, College of Medicine and Health Sciences, United Arab Emirates University, Al Ain, United Arab Emirates; ^3^Department of Radiology, College of Medicine and Health Sciences, United Arab Emirates University, Al Ain, United Arab Emirates; ^4^Department of Genetics and Genomics, College of Medicine and Health Sciences, United Arab Emirates University, Al Ain, United Arab Emirates; ^5^Department of Pharmacology and Therapeutics, College of Medicine and Health Sciences, United Arab Emirates University, Al Ain, United Arab Emirates

**Keywords:** microbiota, dysbiosis, interneuron, gut-brain axis, dendritic morphology

## Abstract

**Introduction:**

A growing body of evidence suggests that the gut microbiome may contribute to changes in brain morphology. The microbiota-gut-brain axis (MGBA) has been shown to influence neurogenesis, axon myelination, and synapse structure. However, it remains unclear whether the MGBA can influence the morphology and density of inhibitory GABAergic interneurons. The aim of this study was to determine whether antibiotic-induced dysbiosis (AID) is associated with alterations in dendritic morphology of GABAergic inhibitory interneurons in the medial entorhinal cortex (mEC), somatosensory cortex (SSC), motor cortex (MC), and hippocampus (Hp).

**Methods:**

A cohort of six-month-old GAD-67-EGFP transgenic mice was treated with an antibiotic cocktail for two weeks, resulting in gut dysbiosis as validated by collecting stool samples at baseline and after treatment, then using next-generation sequencing of 16S ribosomal RNA.

**Results:**

The results demonstrate that the proposed model effectively exhibited the defining features of gut dysbiosis, including a significant reduction in microbiome diversity, expansion of pathobionts, and loss of beneficial microbes. The AID group showed alterations in density and morphology of GABAergic interneurons in different brain areas. The mean dendritic length and mean dendritic segments of the SSC and Hp were found to be significantly decreased, while no such decrease was observed in the mEC or MC. Furthermore, the density of interneurons was decreased in the mEC, Hp, and SSC areas, while no change was observed in the MC area.

**Discussion:**

The interneuron dysfunction plays a role in the pathogenesis of neurological disease. The findings of this study suggest that AID potentially influences the density and morphology of the interneurons, which may contribute to the development of neurological disorders.

## 1 Introduction

The gut microbiota, comprising a vast array of microorganisms that inhabit the gastrointestinal (GI) tract, has the capacity to influence brain function. In contrast to the brain, the gut microbiota is susceptible to direct intervention through the administration of prebiotics, probiotics, and antibiotics, and is responsive to modification by lifestyle factors. The concept of the MGBA emerged from extensive research conducted over the past three decades, which has clearly demonstrated a direct connection between the gut and the brain (Rhee et al., 2009; Cryan and O'Mahony, 2011; De Palma et al., 2014; Cryan et al., 2019; Nakhal et al., 2024). Gut microbiota dysbiosis is defined as a change in the diversity, composition, and function of the gut microbiota (Vangay et al., 2015; Ramirez et al., 2020). Alterations in the microbiota can be attributed to factors intrinsic to the host and/or external to the host, including an unbalanced diet, exposure to pathogens and toxins, long-term use of proton pump inhibitors (PPIs), exposure to antibiotics, excessive alcohol consumption, increased sugar or protein intake, pesticide exposure, poor dental hygiene, and long-term stress (Kesavelu and Jog, 2023). Recently, a variety of tools and techniques have been introduced to facilitate the study of the MGBA, thereby enabling researchers to narrow the gaps in understanding of the MGBA. These include the germ-free mouse model, fecal microbiota transplantation (FMT), probiotics, and prebiotics. Additionally, antibiotic-induced dysbiosis (AID) is a significant factor to consider. An increased exposure to antibiotics during early childhood has been linked to an elevated risk of AID (Duan et al., 2022). This phenomenon is associated with a reduction in the diversity of gut microbial species and abundance of certain taxa, a disruption of host immunity, and the emergence of antibiotic-resistant microbes.

The gut microbiota has been demonstrated to exert influence over a number of processes within the central nervous system, including neurogenesis, myelination, dendritic morphology, microglia morphology, blood-brain barrier structure and permeability, synapse structure and function. A growing body of evidence indicates that microbes within the gut microbiome play a role in brain morphology alterations. Initially, studies conducted on germ-free (GF) animals indicated that the absence of microbiota can influence brain morphology (Hegstrand and Hine, 1986; Sudo et al., 2004; Gareau et al., 2011; Heijtz et al., 2011; Neufeld et al., 2011; Clarke et al., 2013). Secondly, animals that received particular strains of bacteria observed changes in different brain regions (Mckernan et al., 2010; Bercik et al., 2011; Savignac et al., 2014; Desbonnet et al., 2015). Also, human genomic studies on these strains and the brain validated the possible applicability of the findings (Tillisch et al., 2013; Allen et al., 2016; Pinto-Sanchez et al., 2017). Furthermore, population-based research on individuals affected by infection, has showed changes in brain structure and overall microbiota composition (Thabane et al., 2010). Finally, preclinical studies have demonstrated long-term impacts on the brain, spinal cord, and enteric nervous system (ENS) from antibiotic exposure or chronic bacterial infection during early life or adulthood as a result of gut dysbiosis (Verdu et al., 2008; O'Mahony et al., 2014). The gut microbiota is capable of communicating with the brain in a number of ways, including the use of neuronal pathways and small molecule messaging systems. Nevertheless, further research is required to gain a comprehensive understanding of the influence of bacteria in the GI tract on brain function and behavior. Signals generated in the gut can be transmitted to the brain through various pathways (Guzzetta et al., 2022; Kasarello et al., 2023). The primary mode of immune communication is the release of cytokines by immune cells into the circulation. Additionally, pathogen-associated or damage-associated molecular patterns may enter the circulation and affect the functioning of internal organs and the gut microbiota. Moreover, endocrine communication represents the most expansive form, encompassing the hypothalamic-pituitary-adrenal axis (HPA). Neural communication is primarily dependent on direct anatomical connections established by the vagus nerve or indirect connections facilitated by the ENS. Despite substantial evidence indicating a correlation between the vagus nerve and microbiome-to-brain signaling, the neuronal networks underlying the MGBA remain largely unelucidated. Further research is imperative to elucidate these circuits.

The dendrites of neurons represent the principal input compartment, and the optimal growth and arborisation of dendrites are vital for the optimal functioning of the central nervous system. The dendrites of a neuron contain a variety of receptors that are designed to receive signal input from other cells for the purposes of communication, differentiation and maturation. Defects in the development of dendrites impair the formation of neuronal circuits and the processing of information between neurons. The mechanisms regulating dendritic growth are controlled by both cell-intrinsic genetic programs and by extrinsic signaling molecules (Jan and Jan, 2003; Schuldiner and Yaron, 2015; Ledda and Paratcha, 2017; Lin et al., 2020; Hamad et al., 2023).

The neocortical inhibitory interneurons constituted 20% of the total neuron population, and their proper function is imperative for maintaining excitatory and inhibitory balance, which is a fundamental component of brain network dynamics (Ben-Ari, 2001). Furthermore, certain neurological disorders have been classified as “interneuropathies” due to the underlying cause of these diseases being a deficiency in inhibition, which can result in hyperexcitable networks (Paterno et al., 2020). The proper function of interneurons is therefore essential for maintaining normal network activity. A growing body of research has demonstrated that a significant number of interneuropathies, including schizophrenia, bipolar disorder, depression, and epilepsy, are associated with altered gut microbiota (Nakhal et al., 2024; Yassin et al., 2025). A comparative analysis of the gut microbiota composition revealed significant differences between individuals with schizophrenia and healthy individuals. For instance, a study have indicated that schizophrenia is associated with alterations in the gut microbiome, chronic gastrointestinal inflammation, and oxidative stress (Nguyen et al., 2019). Therefore, it is imperative to investigate the impact of gut dysbiosis on the morphology of interneurons in the adult brain. In view of the aforementioned evidence, the present study aims to ascertain whether there is a potential influence of gut microbiota on the morphology and density of inhibitory GABAergic interneurons in the brain.

While the intestinal flora of the mother can influence fetal brain development through placental mechanisms, our study focuses on the impact of the animal's own intestinal flora at 6 months of age. At this stage, the microbiota of the animal has been fully established and is responsible for modulating brain function via mechanisms such as gut-brain axis signaling and microbial metabolites. Thus, our findings are centered on the postnatal microbiota.

## 2 Materials and methods

### 2.1 Ethics statement

All mouse experiments were reviewed and approved by the local ethic commission. License for animal experiments has been obtained from the United Arab Emirates University Animal Ethics Committee of the United Arab Emirates University under the Permission Number: ERA_2023_3752.

### 2.2 Animals and AID procedure

**Figure 1:**
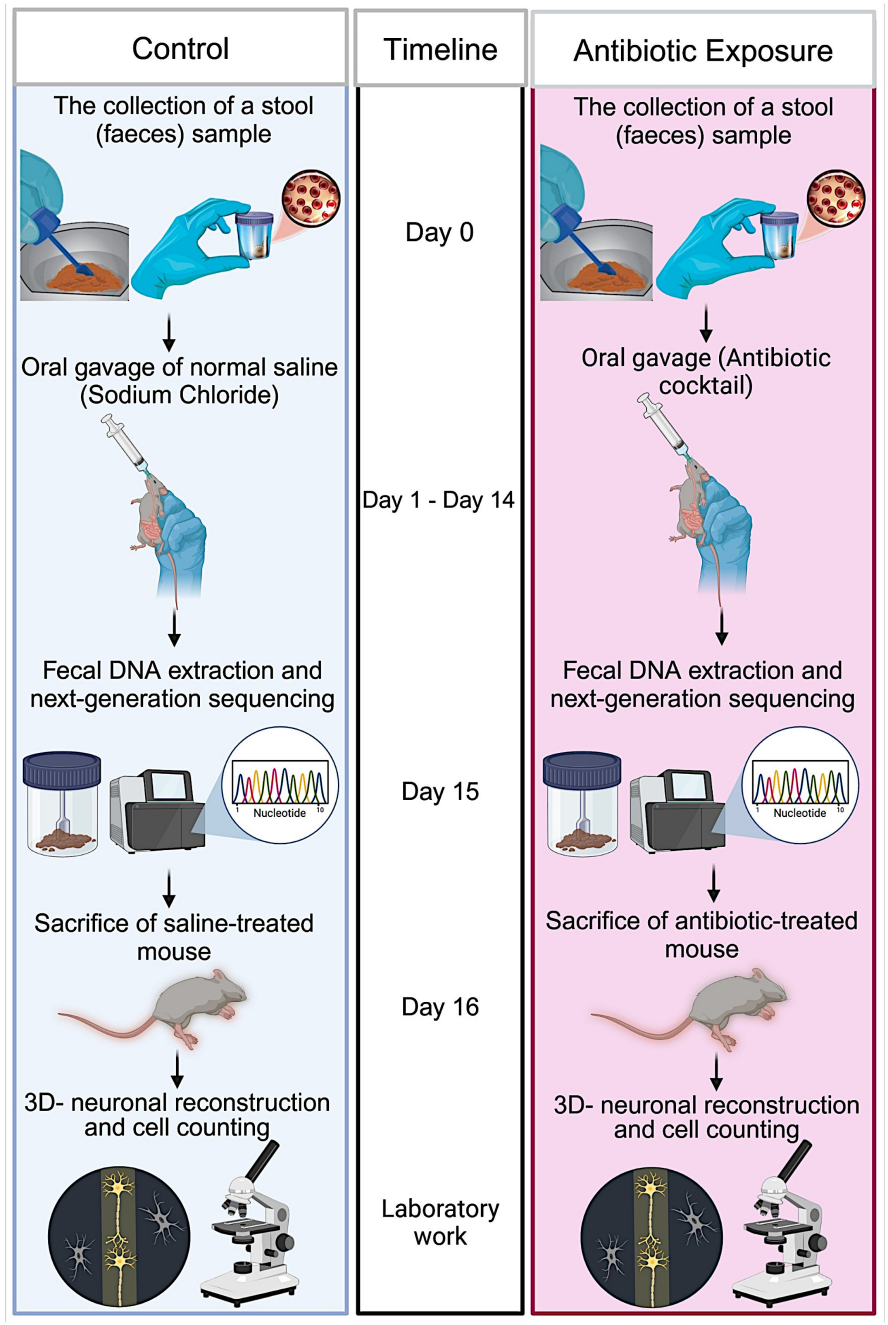
Schematic representation of the experimental procedure. Adult 6-month-old male GAD67-EGFP mice were placed together in a standard plexiglass cage within a temperature-controlled room. On day 0, fecal samples were collected from both animals in the study group. The mice were administered a freshly prepared mixture of blood-brain barrier impermeant antibiotics, containing vancomycin, clindamycin, and meropenem, or vehicle (only H_2_O) via oral gavage once daily for 14 consecutive days. On day 15, fecal samples were collected from both groups following the conclusion of the antibiotic treatment. The DNA was isolated from the fecal samples in accordance with aseptic procedures. The 16S rRNA gene was amplified using the 16S Barcoding kit, and the amplified DNA was sequenced using next-generation sequencing technology. On day 16, the mice were anesthetized and transcardially perfused. The brains were removed, sliced, and subjected to immunohistochemistry. Three-dimensional neuronal reconstructions were performed, and the neuronal morphology of labeled interneurons was quantified.

Experimental procedure is summarized in Figure 1. Animals were housed in a standard 12-h light cycle and fed *ad libitum* with standard mouse chow (Rodent Breeder Feed 24% Pellet, Fujairah Feed Factory, UAE). The genetic background of GAD67-EGFP mice is described in Oliva et al. (2000). As breeders, a male and three nulliparous 6-month-old GAD67-EGFP mice (Oliva et al., 2000) were placed together in a standard plexiglass cage in a temperature-controlled room. Water and food were made available *ad libitum*. From three litters, we selected only male mice at age of 6 months. We selected only males for these experiments to rule out the effects of hormonal changes. These litters were counterbalanced fairly across the control and treatment groups to minimize any potential litter-specific effects. The experimental group (*n* = 6) received a freshly prepared mixture of blood-brain barrier impermeant antibiotics, containing 1.6 mg/ml of vancomycin (Vancolon, Julphar, UAE), 0.83 mg/ml of clindamycin (Vianex S.A, Greece), and 4.8 mg/ml of meropenem (Meronem, Pfizer, USA). The control group (*n* = 6) received vehicle (normal saline). Mice were administered 0.15 mL of the antibiotic mixture or vehicle via oral gavage once daily for 14 consecutive days in accordance with the Washington State University Institutional Animal Care and Use Committee protocol (Turner et al., 2011). Fecal samples were collected the by restraining the animal, taking care not to cause any stress or pain to the animals. Fecal samples were collected from both groups on Day 0, prior to treatment, and on Day 15, following treatment. The selection of antibiotic cocktail was based on comparable research in mice and rats which have previously employed similar treatment procedures, resulting in the development of substantial alterations in the microbiome (Soares et al., 2017; Xie et al., 2019; Hertz et al., 2020; Lee et al., 2020). Throughout the manuscript, the following abbreviations are employed for the various animal groups: Pre-C: control animal group before treatment; Post-C: control animal group after 14 days of vehicle (normal saline) treatment; Pre-T: antibiotic-treated animal group before treatment; Post-T: 14 days of antibiotic-treated animals.

### 2.3 DNA extraction and quantification

DNA was isolated from the fecal samples under aseptic conditions using the QIAamp PowerFecal Pro DNA kit (Qiagen, Hilden, Germany) in accordance with the manufacturer's instructions. The concentration was then measured by Qubit 4 fluorometer (Thermo Fisher Scientific, Waltham, MA, USA) utilizing the Qubit 1X dsDNA High sensitivity assay kit (Invitrogen, Life Technologies Corporation, Eugene, OR, USA).

### 2.4 Nanopore sequencing

The 16s rRNA gene (1.5 kb) was amplified using the 16S Barcoding kit 24 V14 SQK-16S114.24 (Oxford Nanopore Technologies, Oxford, UK) and LongAmp Hot Start Taq 2X Master mix (New England Biolabs, UK) with input of 10 ng genomic DNA per sample. A volume of 10 μL of each 16S Barcode was transferred into respective sample containing tubes. DNA amplification was performed by LifeECO thermocycler (Bioer, Hangzhou, China) with specific cycling conditions and the amplicons were quantified using a Qubit fluorometer. The barcoded samples were then pooled in an equimolar concentration and purified using AMPure XP Beads, followed by quality control check. A quantity of 50 fmol of the pooled sample was used for purpose of the library preparation. The sequencing was performed on a minION MK1C (Oxford Nanopore Technologies, Oxford, UK) device using R10.4.1 flow cell FLO-MIN114 (Oxford Nanopore Technologies, Oxford, UK) in accordance with the manufacturer's protocol for a duration of ~48 h (Elzayat et al., 2023; Rahman et al., 2023).

### 2.5 Bioinformatics analysis

The minKNOW version 6.0.11 (Oxford Nanopore Technologies, Oxford, UK) was used for real time analysis of base calling and data acquisition with a minimum Q score of 9. The basecalled reads were stored in FASTQ files. Post run analysis was performed on EPI2ME software (Oxford Nanopore Technologies, Oxford, UK). The sequences were assigned taxonomy using Kraken2 and Silva in order to ensure the highest degree of accuracy. EPI2ME generated a taxonomic abundance table, which plotted the relative abundance and effect size of bacterial families and genera as well as the richness of species. The operational taxonomic unit (OUT) generated was subsequently subjected to further downstream analysis via Microbiome Analyst 2.0 (McGill, Canada) where linear discriminant analysis effect size (Lefse) plots, alpha diversity and beta diversity were studied (Chong et al., 2020; Elzayat et al., 2023; Lu et al., 2023).

### 2.6 Statistical analysis for NGS experiments

All the statistical analyses were performed using the MDP module in the Microbiome Analyst 2.0 in accordance with an established protocol (Masad et al., 2024). The data were filtered with a minimum count of 0 and a prevalence filter of 20% for the low count filter. The filtered count data were then normalized using Total Sum Scaling (TSS). Core microbiome analysis at the species level was examined using a heatmap generated by 20% sample prevalence and 0.01 relative abundance. Diversity indices were presented as a box plot. Alpha diversity measures (Chao1, and Simpson) were compared using *post-hoc* pairwise comparison for multiple groups by Welch *t*-tests/ANOVA. Beta diversity study was conducted using the Bray-Curtis index to assess the dissimilarity among the groups and it was visualized by principal component analysis (PCA). Permutation-Based Analysis of Variance (PERMANOVA) was employed as a statistical tool to compare beta diversity indices across four different groups, with a significance threshold set at a *P*-value of 0.05 or less. The Linear Discriminant Analysis (LDA) was used to evaluate the relevance or effect size of differentially abundant features. The threshold for the logarithmic LDA score of the discriminative features was at 2 (Dhariwal et al., 2017; Chong et al., 2020, p. 202; Elzayat et al., 2023; Lu et al., 2023; Rahman et al., 2023).

### 2.7 Animal transcardial perfusion and immunohistochemistry

By day 16 of the experiment, the microbiota modulation would have likely had sufficient time to influence the brain, particularly in aspects such as dendritic morphology and interneuron density. This time point was ideal for examining the morphological consequences of microbiota manipulation in an adult brain, which is relevant for understanding how the gut microbiota affects existing, developed neural networks. The animals were deeply anesthetized with 5% isoflurane and perfused transcardially with phosphate-buffered saline (PBS), followed by a fixative containing 4% paraformaldehyde in 0.1 M phosphate buffer (pH 7.2). Immediately following perfusion, the brains were removed and postfixed overnight in the same fixative. They were then cryoprotected overnight in 30% sucrose in phosphate-buffered saline (pH 7.4). Subsequently, a vibratome was utilized to obtain 150 μm parasagittal sections from both hemispheres of the lateral entorhinal cortex, extending to the midline. The Allen Brain Atlas was employed as a reference for the collection of brain slices from the mEC, Hp, SSC, and MC. Subsequently, an immunohistochemistry procedure was conducted using the anti-EGFP antibody to convert EGFP into a stable visible 3,3′-diaminobenzidine (DAB) staining, thereby enhancing resolution for three-dimensional reconstruction. Subsequently, the brain slices were washed several times with TBS (Tris-buffered saline: 50 mM Tris, 150 mM NaCl, pH 7.6) and permeabilized in TBST (TBS, 0.1% Triton X). They were then blocked for 1 h with 1% normal goat serum in TBST. The brain slices were incubated for 24 h at room temperature with the primary antibody, chicken anti-GFP (1:8000, Abcam, ab13970). Subsequently, the secondary antibodies were added in accordance with the manufacturer's instructions, following two washes in TBS. Subsequently, the rabbit anti-chicken biotinylated antibody (1:300, Cat# E043201-8, Dako) was applied for 3 h at room temperature. Following this, the slices were washed several times in TBS buffer. The ABC-horseradish peroxidase method was then employed using diaminobenzidine as the chromogen.

### 2.8 GAD67-positive interneurons counting

Quantitative analyses were conducted on parasagittal brain serial sections (150 μm) to define the borders of the mEC, SSC, MC, and Hp regions. The profile density of GAD67-positive interneurons was estimated by counting the immunolabeled (with anti-EGFP antibody) within the delineated region of interest (ROI). Images of the cell body area were captured on a light microscope (Zeiss, Germany) with a 40 × objective using a 1 mm^2^ grid. The images were then analyzed in MacBiophotonics software.

### 2.9 Three-D neuron reconstruction of interneurons

EGFP-immunostained interneurons were reconstructed with the Neurolucida system (MicroBrightField) at 1000× magnification. To quantify the morphology of GAD67-positive interneurons, a series of parameters were calculated. These included the mean dendritic length (total dendritic length divided by the number of primary dendrites), the mean number of dendritic segments (total number of dendritic segments divided by the number of primary dendrites), and the number of primary dendrites. In order to reconstruct the three-dimensional anatomy of the brain, the following regions were considered: the medial entorhinal cortex (mEC), the subiculum (SSC), the medial cortex (MC), and the hippocampus (Hp). A Sholl analysis was conducted to identify the area where dendritic complexity changed. This was achieved by examining the number of dendrite intersections at 10 μm interval distance points, starting from the cell soma (Sholl, 1953; Zagrebelsky et al., 2010; Hamad et al., 2011, 2014).

### 2.10 Statistical analysis for morphological analyses

The statistical analyses were conducted using Sigma Stat 12 (SPSS Incorporated). Comparisons between two groups were conducted using either the Student's unpaired *t*-test or the Mann-Whitney test, depending on whether the normality test (Shapiro-Wilk) passed. In cases where more than two groups were being compared, a one-way ANOVA was employed, with a Holm-Sidak multiple comparison test used for *post-hoc* analysis if the normality test passed. In the event that the normality test was unsuccessful, a one-way ANOVA on ranks was conducted, followed by a Tukey multiple comparison test for *post-hoc* analysis. This was employed to identify the significant groups. The results were deemed statistically significant at the *p* < 0.05 level. For the Sholl dendritic intersection analyses, we performed a 2-way repeated measures ANOVA with treatment (dysbiosis vs. control) as the between-group factor and radial distance from the soma. Violation of the sphericity assumption for repeated measures was corrected using the Greenhouse-Geisser correction for degrees of freedom. At each distance interval between the control and AID groups, *t*-tests *post-hoc* with Bonferroni correction were performed.

## 3 Results

### 3.1 AID diminishes colonization resistance which allows pathobionts to thrive

**Figure 2:**
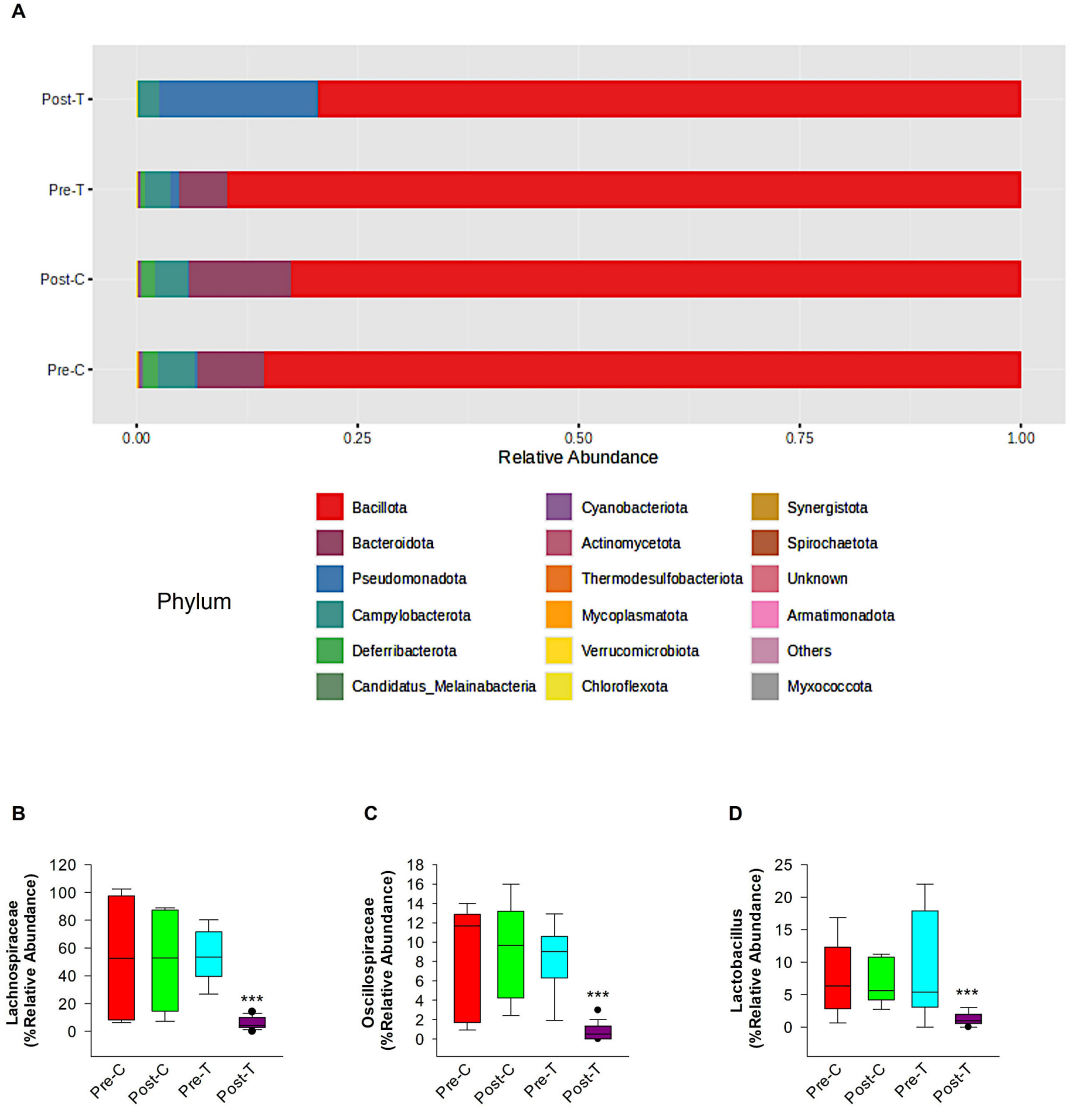
Relative abundance of phylum-level taxa, SCFA-producing bacteria families and lactobacillus genus in 6-month-old GAD67-GFP mice among control and treatment groups. This figure depicts the comparative abundance of bacterial taxa at the phylum level among four experimental groups: Pre-C, Post-C, Pre-T, and Post-T. **(A)** The data is illustrated by stacked bar charts, where each bar signifies the percentage abundance of distinct phyla within each category. Panel comparisons illustrate the alterations in microbial composition between baseline (Pre-C, Pre-T) and post-treatment (Post-C, Post-T) settings. The Post-T group, administered an antibiotic cocktail to induce gut dysbiosis, exhibits a marked reduction in phyla diversity, as indicated by the decreased number of dominant phyla and overall diminished microbial complexity relative to the other groups. statistical analysis and figures were obtained from MicrobiomeAnalyst. The short-chain fatty acids (SCFAs) producing families' relative abundance of *Lachnospiraceae* **(B)** and *Oscillospiraceae* **(C)** and relative abundance of the beneficial *Lactobacillus* genus **(D)**. Relative abundance data were obtained from Epi2me 2.0. PERMANOVA; ***P < 0.001.

**Figure 3:**
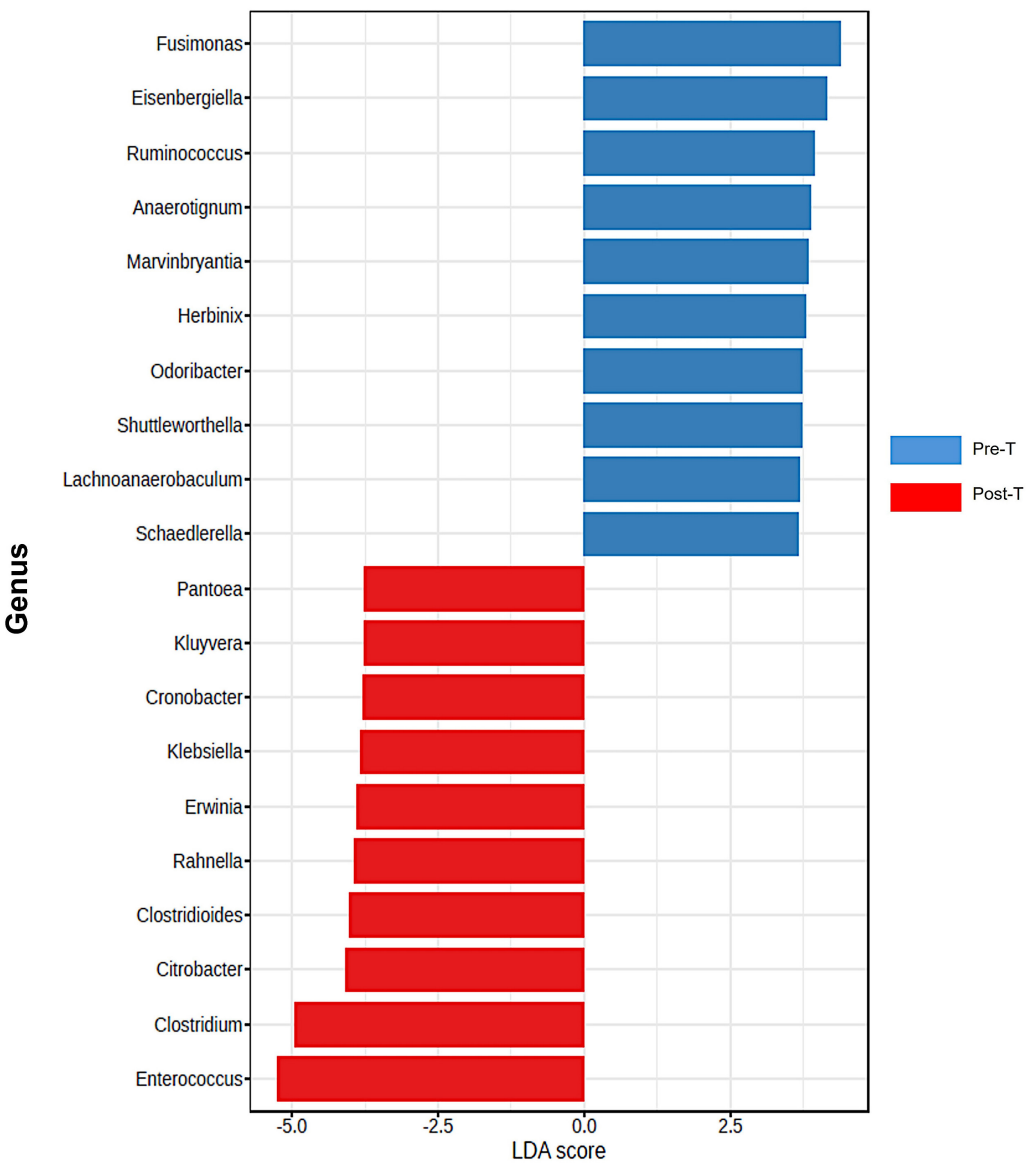
Differentiation of Pre-T and Post-T groups based on LDA scores. Differentially abundant features were identified by using the Kruskal-pretreatment rank-sum test for comparing group distributions with linear discriminant analysis (LDA) to estimate the effect size of the features. analysis of top discriminative bacteria genera between gut samples from pre-treatment and post-treatment (*n* = 6) samples, which emphasized a notable expansion of harmful genera (*P* < 0.05 for all pairwise comparisons). statistical analysis and figures were obtained from MicrobiomeAnalyst.

**Figure 4:**
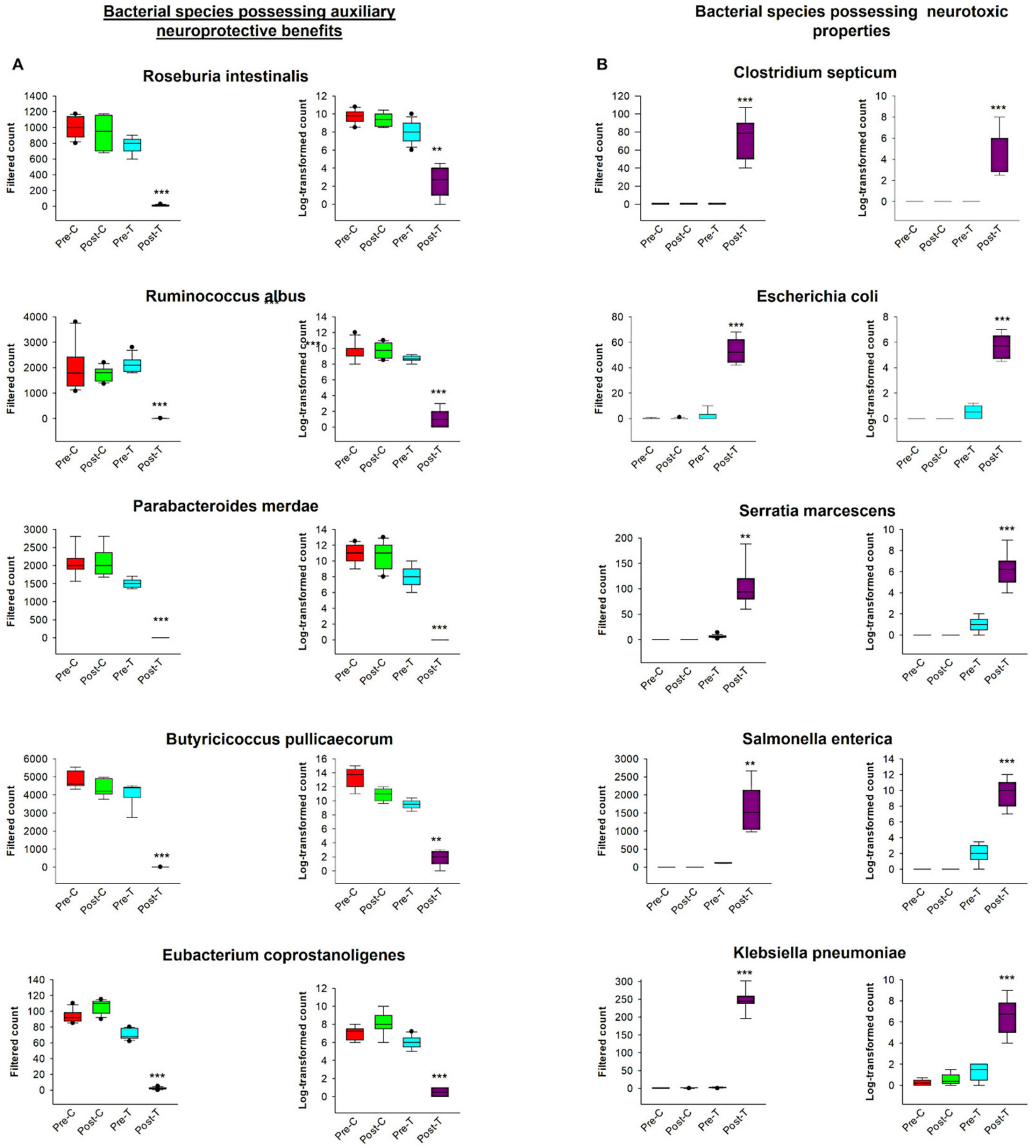
Comparative analysis of bacterial species exhibiting auxiliary neuroprotective benefits and neurotoxic properties. The graphs contained filtered and log-transformed counts across measured groups. **(A)** Illustrates the filtered counts and log-transformed counts of five bacterial species exhibiting neuroprotective advantages. Data for each species is categorized into four experimental groups: Pre-C, Post-C, Pre-T, and Post-T. The box plots illustrate the distribution of bacterial counts across each group. **(B)** Displays the filtered counts and log-transformed counts of five bacterial species recognized for their neurotoxic characteristics. The data is categorized into the same four experimental groups (Pre-C, Post-C, Pre-T, and Post-T), illustrating the variation in the abundance of neurotoxic species across distinct temporal points and conditions. These graphs provide a direct comparison of microbiota dynamics about their distinct neuroprotective or neurotoxic functions to comprehend the effect AID. Filtered and log-transformed counts data were obtained from MicrobiomAnalyst. PERMANOVA, ***P < 0.001; **P < 0.01.

According to the comparative abundance of bacterial taxa at the phylum level (Figure 2A), animals treated with antibiotics showed remarkably decreased levels of *Bacteroidota* in comparison with healthy animals, knowing that *Bacteroidota* is part of the normal flora found in the intestines and the microbial ecosystem (Colella et al., 2023). Furthermore, *Pseudomonadota* phylum which is known to dominate microbial community in high levels of intestinal inflammation (Wang Y. et al., 2023), has significantly increased in the Post-T group as a result of dysbiosis when compared with other groups, leading to imbalance in the microbial community. While *Bacillota* phylum displayed no significant changes among all groups. Overall phylum analysis displayed major shifts after treatment with antibiotics cocktail. Short-chain fatty acids (SCFA's) producing families, such as *Lachnospiraceae* (Figure 2B) and *Oscillospiraceae* (Figure 2C), were reduced remarkably when compared with the Pre-T and other control groups. Moreover, the beneficial microbial *Lactobacillus* genus was found to be significantly reduced in the Post-T group (Figure 2D). The identification of differentially abundant features was conducted through the utilization of the Kruskal-Wallis rank-sum test, which was employed for the comparison of group distributions. This was followed by the application of Linear Discriminant Analysis (LDA), which was used to estimate the effect size of the aforementioned features (Figure 3). This revealed that, genera that previously associated with pathobiont expansion, including *Enterococcus, Clostridium, Citrobacter, Clostridioides, Rahnella, Erwinia, Klebsilla, Cronobacter, Kluyvera*, and *Pantoea* exhibited a significant increase following dysbiosis, when compared with the Pre-T group. While the Pre-C, Post-C, and Pre-T LDA scores didn't report any significant variations between them (data are not shown). Bacterial species which are part of the normal flora such as *Lactobacillus taiwanensis, Clostridium hylemonae, Limosilactobacillus reuteri, Marvinbryantia formatexigens, Acetatifactor muris, Eisenbergiella tayi, Anaerotignum aminovorans* (Rey et al., 2010; Amir et al., 2014; Ueki et al., 2017; Hsieh et al., 2018; Lin et al., 2022; Hu et al., 2023; Wang M. et al., 2023; Mandal et al., 2024) were mutually abundant across Pre-C, Post-C, and Pre-T groups (Supplementary Figures S1A–C). On the other hand, they were drastically diminished after AID. Thus, pathobionts such as *Enterococcus canis, Enterococcus hermanniensis, Enterococcus faecium, Enterococcus sulfureus, Enterococcus dispar, Enterococcus cecorum, Enterococcus saigonensis, Clostridium tertium, Citrobacter koseri* (Yuan et al., 2019; Saad et al., 2022; Xu et al., 2024) were the most abundant species in Post-T group (Supplementary Figure S1D). Moreover, bacterial species with neuroprotective properties such as *Roseburia intestinalis, Ruminococcus albus, Parabacteroides merdae, Butyricicoccus_pullicaecorum*, and *Eubacterium_coprostanoligenes* (Park et al., 2017; Dooling and Costa-Mattioli, 2018; Avagliano et al., 2022; Bai et al., 2024; Nakhal et al., 2024; Sun et al., 2024) were notably more less abundant in the Post-T group in comparison to the other groups (Figure 4A). On the other hand, bacterial species with neurotoxic properties such as *Clostridium septicum, Escherichia coli, Serratia marcescens, Salmonella enterica*, and *Klebsiella pneumoniae* (Knapp et al., 2010; Wu et al., 2013; Liu et al., 2018; Galán, 2021; Park et al., 2024) were significantly more abundant among the Post-T group in comparison to other 3 experimental animal groups (Figure 4B).

### 3.2 Antibiotic-induced gut dysbiosis is associated with a reduction in microbial diversity

**Figure 5:**
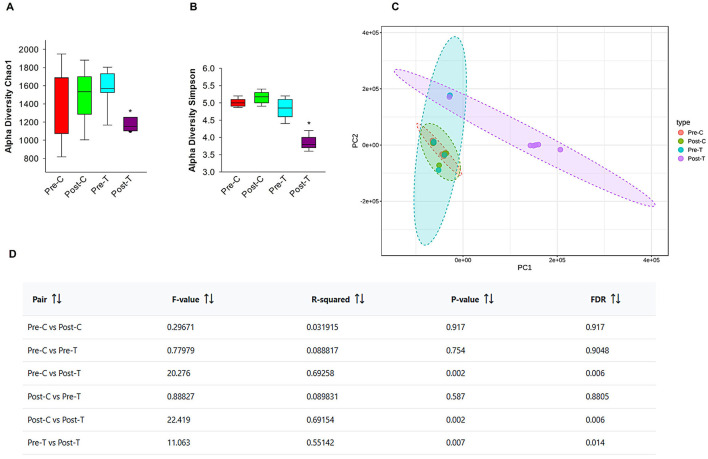
Oral administration of antibiotics decreases microbial diversity in 6-month-old GAD67-GFP mice. Alpha diversity metrics, Chao-1 index **(A)**, Simpson Index **(B)** data were obtained from Epi2me 2.0. *Post-hoc* pairwise comparison (Mann-Whitney test; *P < 0.05). Beta diversity PCA plots **(C)** and beta diversity results table **(D)** were illustrated with and without antibiotic treatment, using MicrobiomeAnalyst. Pairwise PERMANOVA analyses based on Benjamini-Hochberg procedure (FDR).

The evidence is mounting that there is a bidirectional relationship between AID and changes in brain functions and in animal models and humans. These changes occur through various mechanisms (Cryan et al., 2019; Nakhal et al., 2024). To assess alterations in gut microbiome diversity and composition in control and antibiotic-treated subjects and validate the dysbiosis, we conducted next-generation sequencing using fecal samples from mice, which were collected before and after a 14-day course of antibiotic treatment. The compositional diversity of the gut microbiome was evaluated using both alpha and beta diversity metrics. Following a 14-day course of antibiotics, there was a notable decline in richness when compared with the samples obtained prior to treatment and the control group, which received normal saline via the same route of administration as measured by Chao-1 (Figure 5A). Sample evenness, which is another component of alpha diversity, was assessed by employing Simpson Diversity Index (Figure 5B) which revealed a notable reduction post-antibiotic treatment in all other groups. As a reduction in alpha diversity is a key indicator of gut dysbiosis, these findings suggest that the Post-T group meets the criteria for this condition. In contrast to Post-T group, the post control group (Post-C) which received normal saline for 14 days via oral gavage, did not exhibit any remarkable decreases in the gut microbial alpha diversity measures in comparison to pre control group (Pre-C) and pretreatment group (Pre-T). This result eliminates route of administration stress consideration as a confounding variable and indicates that alpha diversity reduction was solely as a result of antibiotic-induced gut dysbiosis.

Beta diversity, an indicator of inter-sample diversity and a measure of group dissimilarity was evaluated using the Bray-Curtis distance metric and displayed by principal component analysis (PCA) (Figure 5C). The results of pairwise PERMANOVA analysis using the multi testing adjustment based on Benjamini-Hochberg procedure demonstrated distinct clustering of the gut microbiota between post-antibiotic treatment Post-T group and all the other groups (Figure 5D), indicating that the change in the overarching microbial composition of the gut occurred after antibiotics' administration. While other groups (Pre-C, Post-C, Pre-T) didn't record any significant statistical difference in beta diversity. These findings demonstrate that the proposed gut dysbiosis model effectively exhibited the defining characteristics of gut dysbiosis, including a notable reduction in microbiome diversity, an expansion of pathobionts, and a loss of beneficial microbes. These observations were made in comparison to untreated control groups or groups that underwent the same stress factor.

### 3.3 AID reduces GAD67-positive interneurons in adult mEC, SSC, and Hp

**Figure 6:**
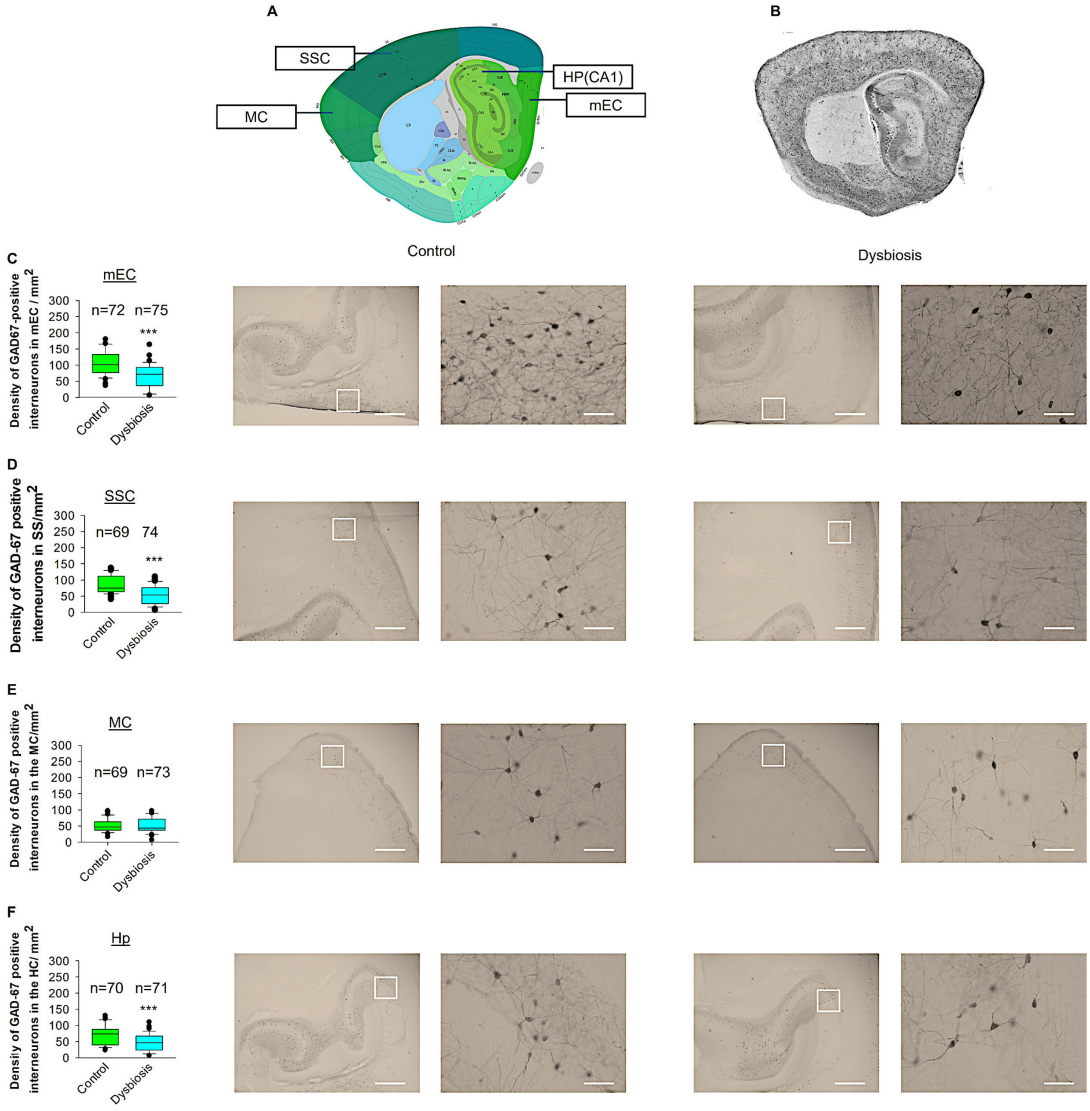
Effect of AID on GAD67-positive interneuron density. **(A)** Virtual parasagittal brain slice from the Allen Brain Atlas reference, illustrating the four principal regions under examination. **(B)** An exemplar of a parasagittal brain slice from a GAD67-GFP-stained brain slice. **(C–F)** The median value is represented by the horizontal lines within the box plots, while the variabilities outside the upper and lower quartiles are indicated with whiskers. The central portion of the data set is illustrated with a box plot. **(C)** Mean values of the number of GAD67-positive interneurons per 1 mm^2^ in the mEC region. The accompanying illustration depicts a photomicrograph captured at 4× magnification (scale bar = 200 μm) and a zoomed area captured at 40× magnification (scale bar = 20 μm) from control and AID mice from the mEC region. It is presented in conjunction with the corresponding graph. *N* = 72 ROI from 12 control mice and *N* = 75 ROI from 12 AID mice. **(D)** The box plot in the graph represents the mean values of the number of GAD67-positive interneurons per 1 mm^2^ in the SSC region. *N* = 69 ROI from 12 control mice and *N* = 74 ROI from 12 AID mice. **(E)** Mean values of the number of GAD67-positive interneurons per 1 mm^2^ in the MC region. *N* = 67 ROI from 12 control mice and *N* = 73 ROI from 12 AID mice. **(F)** Mean values of the number of GAD67-positive interneurons per 1 mm^2^ in the Hp region. *N* = 70 ROI from 12 control mice and *N* = 71 ROI from 12 AID mice. Mann-Whitney *U*-test; ***P < 0.001.

To ascertain whether adult dysbiosis affects the density of GAD67-positive interneurons in the neocortex, we conducted profile densities (number of GAD67-positive interneuron cells per surface area) on parasagittal slices from both brain hemispheres. The number of GAD67-positive interneurons was counted in the mEC, SSC, MC, and Hp of mice in the dysbiosis group (Post-T), and a comparison was made with the control group (Post-C) (Figure 6). In the mEC, Hp, and SSC, the profile density of GAD67-positive interneurons in dysbiosis brain slices was found to be significantly reduced in comparison to the control group (Figures 6C, D, F). In the MC, no significant difference was observed in the profile density of GAD67-positive interneurons between the dysbiosis brain slices and the control group (Figure 6E). It can be concluded that adult dysbiosis results in a reduction in the number of GAD67-positive interneurons in the mEC, Hp, and SSC, but not in the MC.

### 3.4 AID did not affect interneuron morphology in adult mEC

**Figure 7:**
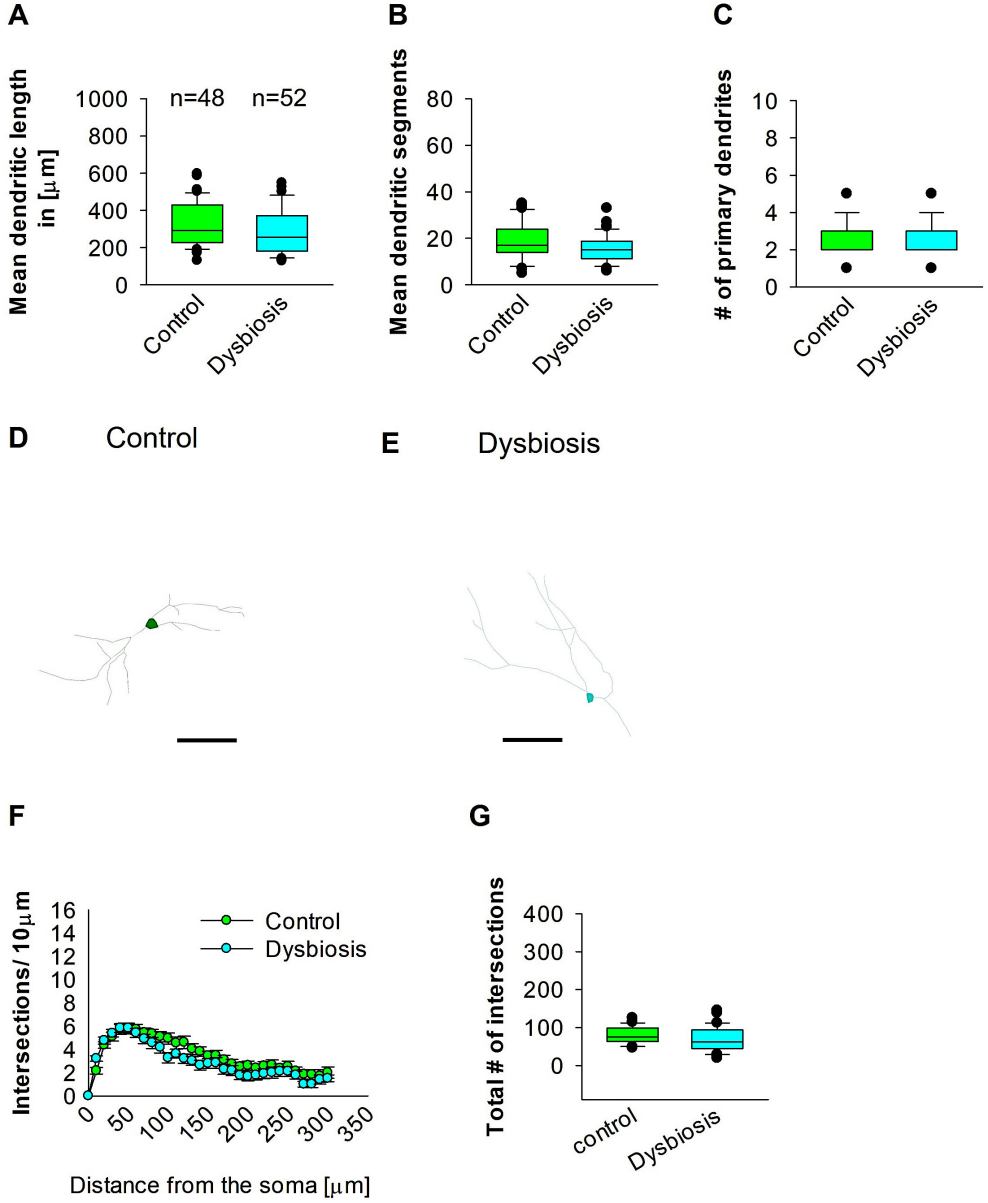
Effect of AID on the morphology of interneuron of the mEC. Parasagittal brain slices from the mEC region were stained with EGFP for visualization of morphology. **(A)** The box plot depicted in the graph represents the mean dendritic length of interneurons in the control and AID groups. **(B)** The box plot in the graph represents the mean values of dendritic segments in the control and AID groups. **(C)** The box plot in the graph represents the mean values of the number of primary dendrites. Representative images at 40× magnification of a control interneuron **(D)** and an AID interneuron are presented **(E)**. The corresponding traces are presented in conjunction with the images. Scale bars are provided in the following images. The scale is μm 50. **(F)** Sholl analysis of the control and AID groups. The error bars in **(F)** represent the standard error mean. **(G)** The box plot depicted in the graph represents the mean values of the total number of dendritic intersections. The total number of cells analyzed for Sholl analyses is presented in **(A)**. The number of reconstructed cells per group is provided above the box plot in **(A)**. *N* = 48 reconstructed cell from 8 control mice and *N* = 52 reconstructed cell from 9 AID mice.

To compare dendritic morphology between AID and control mice, we stained GAD67 mouse brains with anti-EGFP antibody to reconstruct 3D dendritic morphology under light microscopy. Anti-EGFP staining was used to convert the fluorescent dye into a stable DAB stain to prevent bleaching over time. To investigate whether AID influences the morphology of interneurons in adult mEC, we conducted quantitative morphological quantifications. The results of our analyses demonstrated that AID did not affect the dendritic length of GAD67-positive interneurons in adult mEC (Figures 7A, D, E). Similarly, the mean dendritic segments of GAD67-positive interneurons from AID mice were also not significantly different from those of the control group (Figures 7B, D, E). Furthermore, the number of primary dendrites remained unchanged between the two groups (Figures 7C–E). These findings suggest that AID do not exert any influence on the dendritic morphology of interneurons in the adult mouse brain. To confirm that AID does not influence dendritic complexity, we performed Sholl analyses, which demonstrated no significant change in dendritic complexity of the GAD67-positive interneurons in AID animals in comparison to the control group (Figure 7F). Furthermore, the total number of dendritic intersections of interneuron dendrites remained unaltered in AID interneurons (Figure 7G). This data suggests that AID do not affect interneuron morphology in the adult mouse mEC.

### 3.5 Dysbiosis reduces the complexity of the proximal dendritic compartment of hippocampal interneurons

**Figure 8:**
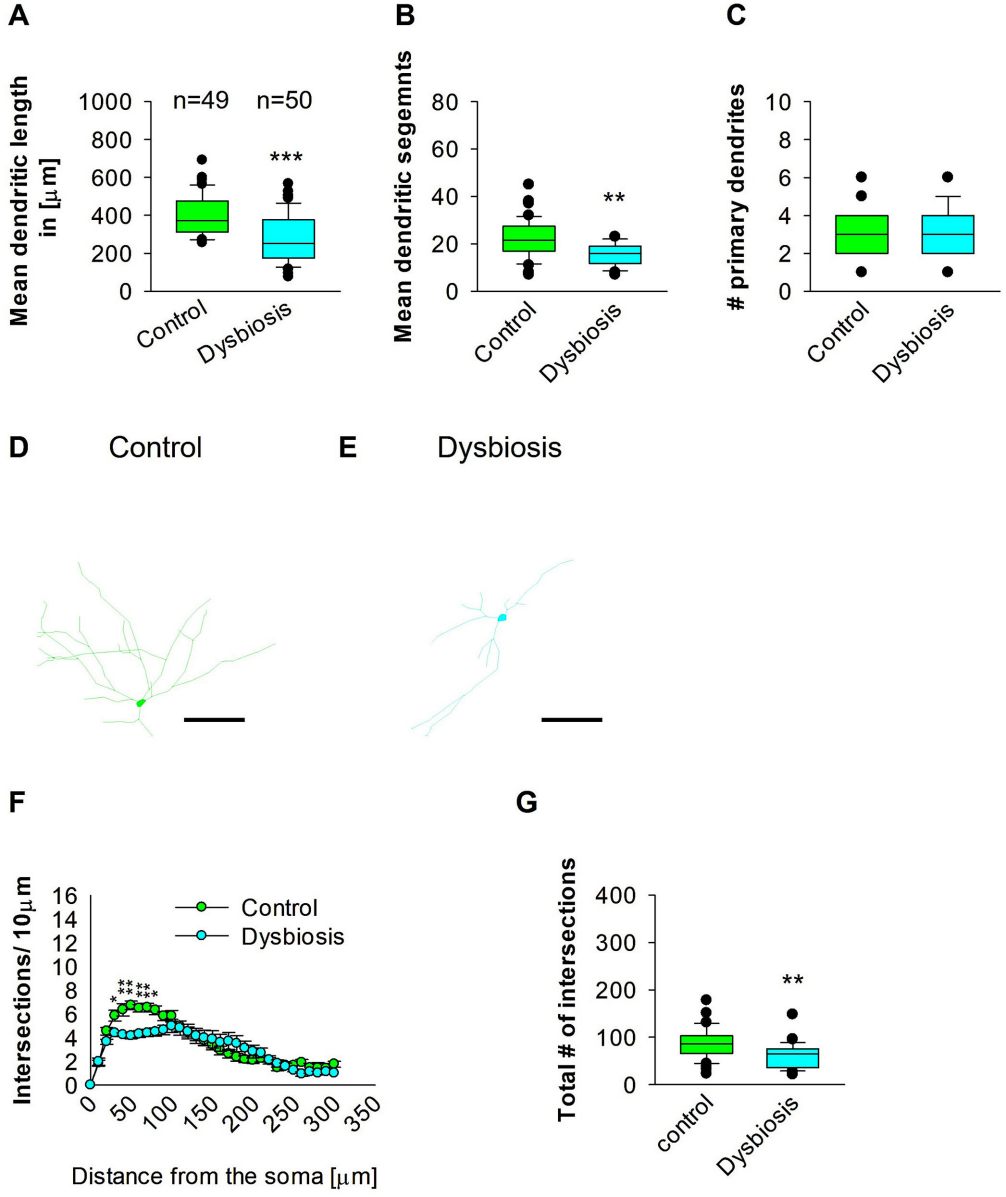
Effect of AID on the morphology of interneuron of the HP. **(A)** The box plot displayed in the graph represents the mean dendritic length of interneurons in the control and AID groups. **(B)** The box plot in the graph represents the mean values of dendritic segments in the control and AID groups. **(C)** The box plot in the graph represents the mean values of the number of primary dendrites. Representative images at 40× magnification of a control interneuron **(D)** and an AID interneuron are presented **(E)**. The corresponding traces are shown next to the images. Scale bars: 50 μm. **(F)** Sholl analysis of the control and AID groups. AID-induced decrease in the number of intersections were observed between 40 and 80 μm from the soma using *t*-test *post-hoc* Bonferroni corrections. *P < 0.05; **P < 0.01. Error bars in **(F)** represent the standard error mean. **(G)** The box plot in the graph represents the mean values of the total number of dendritic intersections. For the graphs in **(A–C, G)**, Mann-Whitney *U*-test; ***P < 0.001; **P < 0.01. The number of reconstructed cells per group is provided above the box plot in **(A)**. *N* = 49 reconstructed cell from 8 control mice and *N* = 50 reconstructed cell from 9 AID mice.

To determine whether AID can influence adult hippocampal GAD67 interneurons, the neuronal three-dimensional reconstructions, followed by quantitative morphological analyses, demonstrated that AID diminishes the dendritic complexity of adult hippocampal interneurons. The mean dendritic length of the AID mice group exhibited a reduction in mean dendritic length (Figures 8A, D, E). Furthermore, the mean dendritic length of GAD67-positive interneurons from AID mice was also found to be decreased in comparison to the interneurons from the control group (Figures 8B, D, E). The number of primary dendrites remained unchanged between the two groups (Figures 8C–E). These findings indicate that AID in adult mice significantly reduces the dendritic complexity of hippocampal interneurons. To ascertain in which dendritic compartment the reduction in dendritic complexity occurs, we conducted Sholl analyses. The analysis demonstrated a notable decline in dendritic complexity at proximal but not distal dendritic intersections of GAD67-positive interneurons from AID mice in comparison to the control group (2-way repeated measures ANOVA, ^***^P < 0.001; Figure 8F). Furthermore, the total number of dendritic intersections of interneuron dendrites was significantly reduced in the AID group (Figure 8G). This data suggests that AID in adult mice results in a reduction in dendritic complexity of proximal dendrites of adult hippocampal interneurons.

### 3.6 AID results in a reduction in the complexity of proximal dendrites of interneurons in the SSC

**Figure 9:**
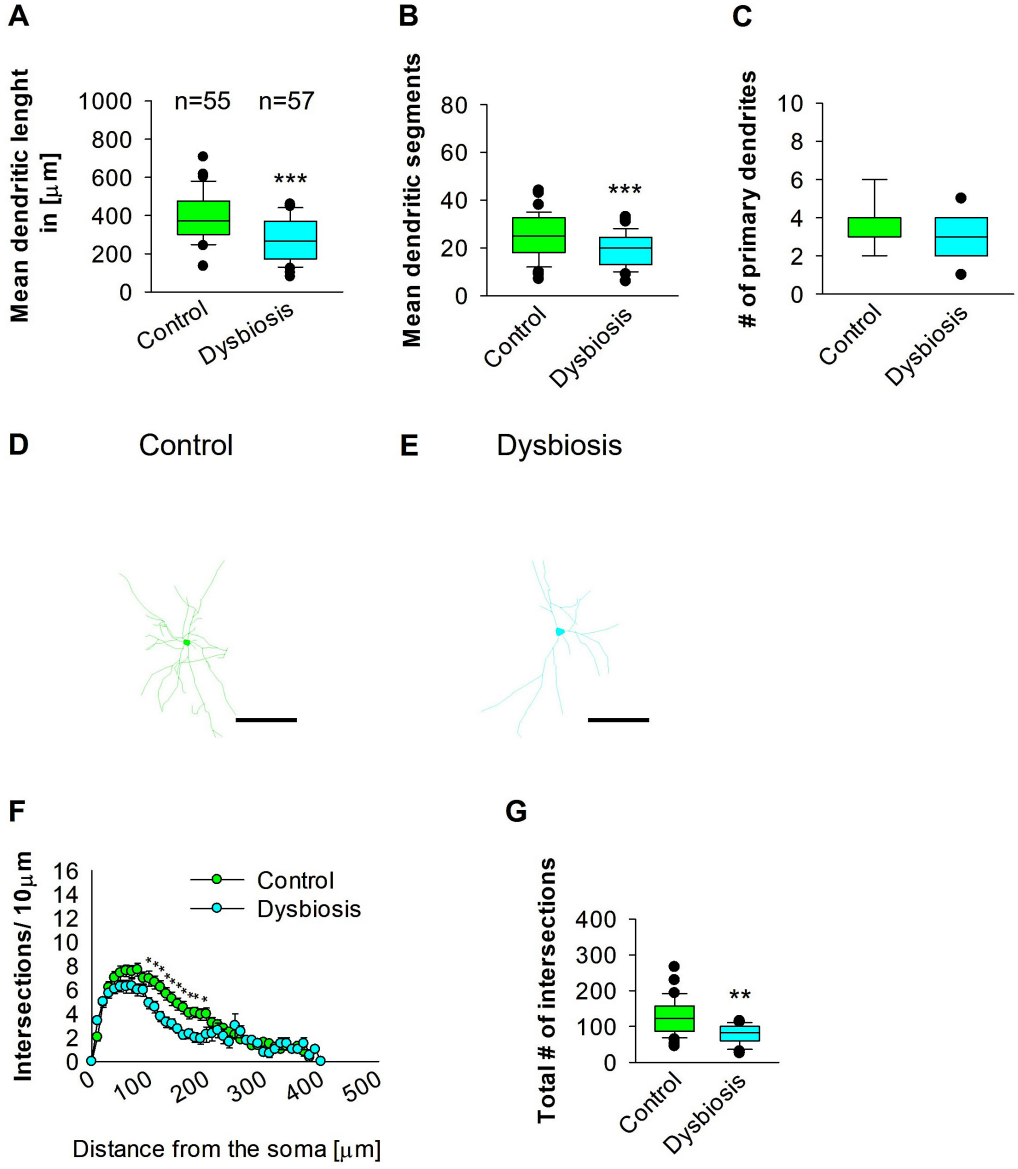
Effect of AID on the morphology of interneuron of the SSC. **(A)** The box plot depicted in the graph represents the mean dendritic length of interneurons in the control and AID groups. **(B)** The box plot in the graph represents the mean values of dendritic segments in the control and AID groups. **(C)** The box plot in the graph represents the mean values of the number of primary dendrites. Representative images at 40× magnification of the control **(D)** and an AID interneuron are shown **(E)**. The corresponding traces are shown next to the images. Scale bars: 50 μm. **(F)** Sholl analysis of the control and AID groups. AID-induced decrease in the number of intersections were observed between 100 and 200 μm from the soma using *t*-test *post-hoc* Bonferroni corrections. *P < 0.05. Error bars in **(F)** represent the standard error mean. **(G)** The box plot in the graph represents the mean values of the total number of dendritic intersections. The number of reconstructed cells per group is provided above the box plot in **(A)**. *N* = 55 reconstructed cell from 8 control mice and *N* = 57 reconstructed cell from 9 AID mice. For the graphs in **(A–C, G)**, Mann-Whitney *U*-test; ***P < 0.001; **P < 0.01.

The objective of the subsequent investigation was to ascertain the impact of AID on the morphology of interneurons in the SSC. To this end, we reconstructed the dendritic arbors of GAD57-positive interneurons from the SSC area. The results of the quantitative morphological analysis demonstrated that AID resulted in a reduction in dendritic length of GAD57-positive interneurons (Figures 9A, D, E). Similarly, the mean dendritic segments of GAD67-positive interneurons from AID mice were also significantly decreased in comparison to the control group (Figures 9B, D, E). The number of primary dendrites remained unchanged between the two groups (Figures 9C–E). These findings indicate that AID has a significant impact on the dendritic morphology of interneurons in the SSC. Subsequently, we employed the Sholl analysis to investigate the specific dendritic compartment of the interneurons in which dendritic complexity was diminished as a consequence of AID. The Sholl analysis revealed a notable decline in dendritic complexity at proximal dendritic intersections of GAD67-positive interneurons in AID-treated animals when compared to the control group (2-way repeated measures ANOVA, ^**^P < 0.01; Figure 9F). Additionally, the total number of dendritic intersections of interneuron dendrites was significantly reduced in AID interneurons (Figure 9G). These findings indicate that AID can induce a growth deficit in interneurons in the adult SSC.

### 3.7 AID did not affect interneuron morphology later in adult MC area

**Figure 10:**
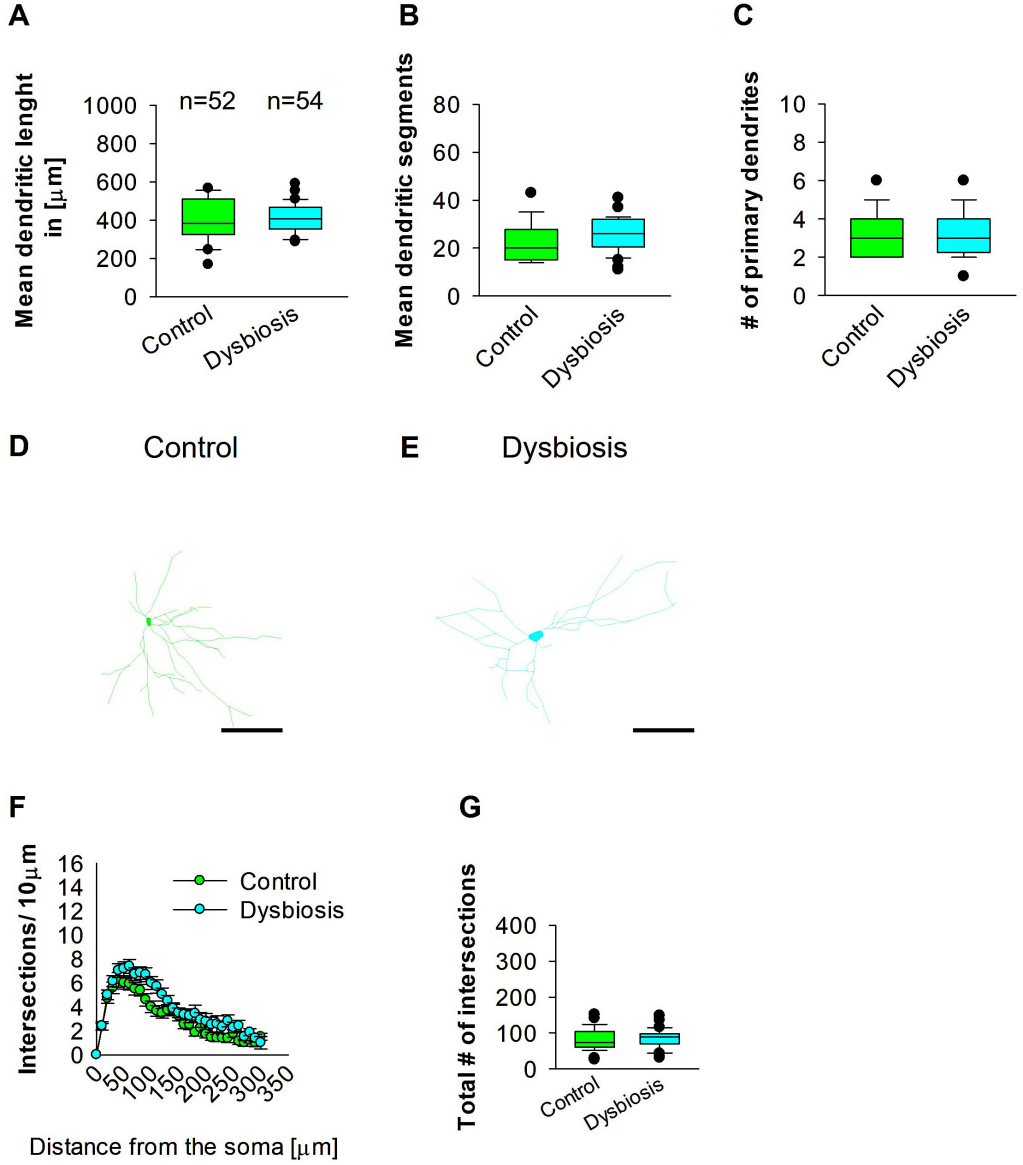
Effect of AID on the morphology of interneuron of the MC. **(A)** The box plot shows the mean dendritic length of interneurons in the control and AID groups. **(B)** The graph shows the mean number of dendritic segments in the control and AID groups. **(C)** The graph shows the mean number of primary dendrites. The number of cells in each group is shown above the box plot in **(A)**. Images of a control interneuron at 40× magnification **(D)** and an AID interneuron **(E)** are shown. The images show the corresponding traces. Scale bars: 50 μm. **(F)** Sholl analysis of the control and AID groups. The error bars represent the standard error mean. **(G)** The box plot represents the mean values of the total number of dendritic intersections. The number of reconstructed cells per group is provided above the box plot in **(A)**. *N* = 52 reconstructed cell from 8 control mice and *N* = 54 reconstructed cell from 9 AID mice.

Next, we investigated whether AID affects interneuron morphology in adult MC. AID did not affect the length of GAD67-positive interneurons in the adult MC of the mouse brain (Figures 10A, D, E). The mean dendritic segments of GAD67-positive interneurons from AID mice were also not significantly different from the control group (Figures 10B, D, E). The number of primary dendrites was the same in the two groups (Figures 10C–E). AID does not affect the morphology of interneurons in adult MC. To confirm that AID do not influence dendritic complexity, Sholl analyses were performed, which demonstrated no significant change in dendritic complexity of the GAD67-positive interneurons in AID in comparison to the control group (Figure 10F). Furthermore, the total number of dendritic intersections of interneuron dendrites was unaltered in the AID interneuron group (Figure 10G). These findings suggest that AID do not affect interneuron morphology in the MC.

## 4 Discussion

The present study examined the impact of AID on the dendritic morphology of GABAergic interneurons in the mEC, SSC, MC, and Hp. The results indicated a reduction in the length and branching of interneurons' dendrites following AID in the SSC and Hp, while no effects were observed in the mEC or MC. This represents a novel finding, as previous studies on AID have not addressed whether AID affects the dendritic morphology of inhibitory interneurons. While our current work focuses on the effects of microbiota disruption, studies in the literature suggest that microbiota restoration can influence brain function and structure, particularly in terms of reversing or modifying changes in neuronal morphology. For instance, restoration of the microbiota in animal models has been shown to partially reverse some of the cognitive and neurophysiological effects caused by microbiota alterations. However, it remains unclear whether dendritic morphology specifically would recover, and further experiments would be needed to examine this. Dendrites of interneurons represent the primary input compartment, which can either receive inhibition from other interneurons or activation from other neighboring excitatory pyramidal cells. The proper growth and development of dendrites are crucial for the proper functioning of the central nervous system. During early development, dendritic growth is regulated by both cell-intrinsic programs and extrinsic factors that regulate various aspects of dendritic development (Jan and Jan, 2003; Lin et al., 2020; Hamad et al., 2023). Over the past three decades, numerous studies have demonstrated that the dendritic growth process is significantly responsive to extrinsic factors, influencing local and global mechanisms of dendrite development (Valnegri et al., 2015). The extrinsic factors include neurotransmitters, neurotrophins, extracellular matrix proteins, contact-mediated ligands, and secreted and diffusible cues. The MGBA has been demonstrated to regulate neuronal dendritic morphology within the brain, and the microbiome has been shown to exert a distinct influence on dendritic structure, either by preserving or altering it. In accordance with prior findings, the administration of the GOS, well-studied prebiotics, for 40 days has been demonstrated to enhance dendritic spine density in rats, a reliable indicator of hippocampal excitatory synapses (Waworuntu et al., 2016). On the other hand, GF mice displayed aberrant mushroom-shaped dendrites that were thinner, shorter, and shrunken in comparison to control mice. This resulted in a reduction in synaptic strength and plasticity, despite an increase in dendritic branching (Ferrante et al., 2013). Furthermore, the mode of delivery affects the composition of the microbiota, which may influence dendritic arborization. Studies have demonstrated that mice and rats delivered by cesarean section have exhibited decreased dendritic arborization or branching (Juárez et al., 2008; Chiesa et al., 2019). The fecal transplantation from aged experimental animals to young recipients has been observed to result in a reduction in dendritic spine density in the hippocampus and prefrontal cortex, as well as impacted memory performance and reduced expression of neuron plasticity proteins (D'Amato et al., 2020; Li et al., 2020). These studies suggest that MGBA can influence the morphology of distinct types of neurons. Moreover, supplementation of a mouse model of amyotrophic lateral sclerosis with GOS as a prebiotic for 74 days has resulted in an increased abundance of the *Bifidobacterium* and *Lactobacillus* genera, thereby reducing motor neuron death and spinal cord inflammation when compared with the untreated group (Song et al., 2013). Moreover, germ-free mice exhibited a reduction pyramidal dendritic length and branching in the hippocampal dentate gyrus and amygdala (London et al., 2013). Taken together, these studies collectively indicate MGBA can impact the dendrite and spine growth of neurons, which may subsequently lead to neurological impairments in adulthood.

While this study provides important insights into the effects of microbiota modulation on neuronal morphology, several limitations should be noted. First, we did not investigate the specific microbial metabolites or active substances responsible for the observed changes in neuronal morphology. Identifying these active substances is a key area for future research. Additionally, our study was conducted at a single time point (day 16) following microbiota disruption, limiting our ability to assess long-term or reversible effects. Future studies should examine multiple time points or investigate the potential for recovery if the microbiota is restored. Second, our study focused exclusively on adult mice, and the effects of microbiota modulation during earlier developmental stages remain unexplored. Further research is needed to assess the impact of microbiota on brain development. Moreover, we did not test the reversibility of dendritic abnormalities through microbiota restoration. This is a critical question that we plan to address in future studies. Finally, while we controlled for major confounding variables, individual differences in microbiota composition or environmental factors may have influenced the results. Future studies with larger sample sizes and more controlled experimental conditions could help mitigate this limitation.

Despite their initial development for the treatment of bacterial infections, antibiotics have become a valuable tool for investigating the impact of these drugs on brain structure and function. In comparison to the GF mice model, antibiotics offer greater temporal flexibility and specificity, as they can be delivered acutely or chronically at any stage across an animal's lifespan. A significant aspect to be taken into account when employing antibiotics for the investigation of MGBA is their absorption from the gastrointestinal tract. Non-absorbable antibiotics (e.g., vancomycin, neomycin, and bacitracin; clindamycin; and meropenem) do not enter the systemic circulation (Tochitani et al., 2016), thus avoiding any potential systemic and even central nervous system effects. This will prevent any potential toxicity resulting from the use of antibiotics, which may otherwise lead to alterations in brain structure and function. No indications of cellular toxicity or necrosis were observed in the analyzed slices. This indicates that the reduction in dendritic length and complexity in the SSC and Hp region resulting from the AID was associated with a reduction in microbial diversity and a decline in colonization resistance, which facilitates the proliferation of pathobionts. Although the blood-brain barrier and intestinal mucosal barrier are physically and physiologically separate, their tight junctions are interconnected, as the release of pro-inflammatory cytokines from the gut, resulting from alterations in intestinal permeability, can communicate with the brain and disrupt the integrity of the blood-brain barrier. Therefore, abundant bacteria species present in gut dysbiosis such as *Clostridium septicum, Escherichia coli, Serratia marcescens, Salmonella enterica*, and *Klebsiella pneumoniae* display neuroinflammatory properties (Knapp et al., 2010; Wu et al., 2013; Liu et al., 2018; Galán, 2021; Park et al., 2024), these findings are consistent with the presented results. Similarly, previous studies emphasized the diminishing of neuroprotective bacteria species in gut dysbiosis model (Marques et al., 2018; Li et al., 2019), which was presented in line with our findings of the depletion of species with neuroprotective properties after gut dysbiosis.

Our results indicate that AID reduces the number of GAD67-positive interneurons in adult mEC, SSC and Hp, but not in MC. Given their structural and functional characteristics, it is not unexpected that interneuron dysfunction plays a role in the pathogenesis of neurological disease states. Indeed, several neurological disorders are now classified as “interneuronopathies”, including schizophrenia, bipolar disorder, depression, and epilepsy (Knopp et al., 2008; Luscher et al., 2011; Chiapponi et al., 2016; Leifeld et al., 2022). Any perturbation in the density or dendritic structure of inhibitory GABAergic interneurons has the potential to lead to pathological hyperexcitability of neuronal circuits, which may contribute to the etiology of disorders such as epilepsy, schizophrenia, bipolar disorder, or autism spectrum disorders (Courchesne and Pierce, 2005; Ishii et al., 2016; Hu et al., 2017). The balance between excitatory and inhibitory processes is a fundamental aspect of brain network dynamics. Indeed, alterations in inhibitory GABAergic neurotransmission, such as reduced or enhanced transmission, have been associated with epileptiform activity or cognitive impairment, respectively (Kalueff and Nutt, 1996; Treiman, 2001). The interneurons control inhibition in these cortical areas, and this suggests that the reduction in GAD67 interneuron density may indicate a change in network activity, which should be more excitable due to the lack of inhibition. To confirm this, future studies will need to perform calcium imaging or patch-clamp recordings to demonstrate that network inhibition is reduced by the reduction in interneuron density in the AID group of animals. Collectively, these data suggest that the reduction in interneuron density caused by AID may indicate an alteration in brain function in the adult mouse brain.

The gut microbiota comprises a diverse population of microorganisms that inhabit the digestive system, which is central to digestion and the immune response. The link between neuroplasticity and the gut microbiota stems from the microbiota's ability to influence brain function and behavior through multiple pathways, including neurotransmitter production, immune modulation, and inflammation regulation. Consequently, this interplay may influence both cognitive function and mental health. Maintaining a balanced gut microbiota is critical for proper gut physiology and MGBA signaling. When there is an imbalance in the microbiota caused by the dysbiosis, it can have adverse effects on several systems, including the GI tract and the CNS. Thus, understanding the mechanisms of synaptic plasticity may provide important insights into the pathophysiology of neuropsychiatric disorders, such as attention-deficit/hyperactivity disorder, and neurological disorders, including epileptic seizures, and point to new therapeutic interventions.

In conclusion, the evidence presented so far suggests that AID can affect interneuron density, morphology in the mouse brain. This study demonstrates that antibiotic treatment has an impact on brain morphology, which can presumably alter brain function. The inhibitory interneurons are critical for maintaining the excitatory/inhibitory balance, highlighting the need to expand research in this area to deepen understanding of the consequences of adverse experiences in the brain.

## Data Availability

The original contributions presented in the study are included in the article/Supplementary material, further inquiries can be directed to the corresponding author.

## Supplementary material

Supplementary Figure 1 Heatmap representation of core microbiome species abundance. This heatmap illustrates the relative abundance of core microbiome species among various groups Pre-C **(A)**, Post-C **(B)**, Pre-T **(C)**, Post-T **(D)** emphasizing the prevalence of pathobionts in contrast to healthy normal flora. The color gradient indicates species abundance, with more abundance depicted in warmer hues. Figures were obtained from MicrobiomeAnalyst.
