# Supplementary figures and images for: Antibiotics-induced dysbiosis impacts dendritic morphology of adult mouse cortical interneurons
Source: Front Neuroanat. 2025 Mar 7;19:1557961. doi: 10.3389/fnana.2025.1557961 (PMC11925899; doi:10.3389/fnana.2025.1557961)

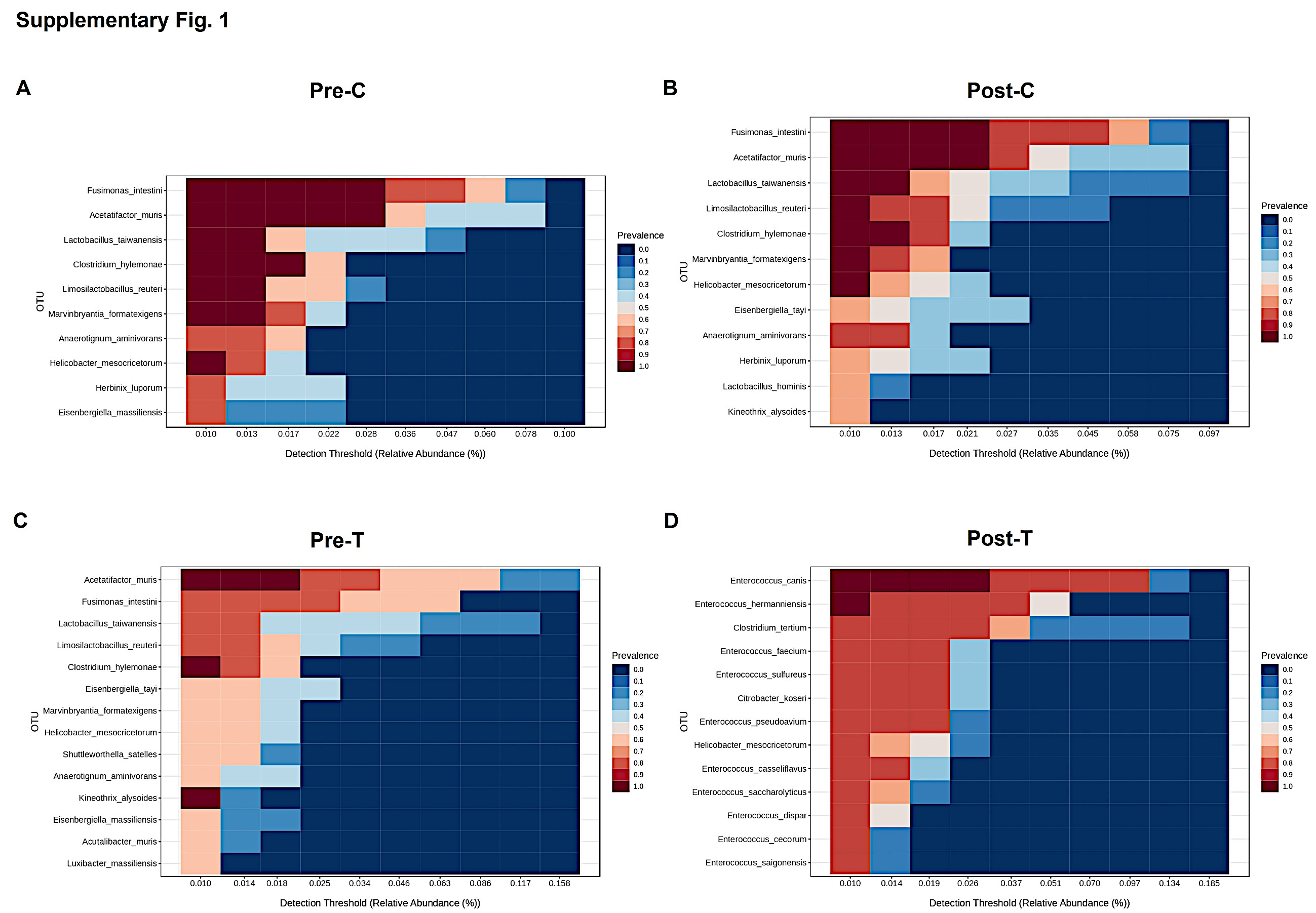

Supplement: Supplementary Figure 1 — Heatmap representation of core microbiome species abundance. This heatmap illustrates the relative abundance of core microbiome species among various groups Pre-C (A), Post-C (B), Pre-T (C), Post-T (D) emphasizing the prevalence of pathobionts in contrast to healthy normal flora. The color gradient indicates species abundance, with more abundance depicted in warmer hues. Figures were obtained from MicrobiomeAnalyst. [file Image_1.jpeg]
